# Prevalence and etiology of false normal aEEG recordings in neonatal hypoxic-ischaemic encephalopathy

**DOI:** 10.1186/1471-2431-13-194

**Published:** 2013-11-22

**Authors:** Gábor Marics, Anna Csekő, Barna Vásárhelyi, Dávid Zakariás, György Schuster, Miklós Szabó

**Affiliations:** 1 First Department of Pediatrics, Semmelweis University, Budapest, Bókay u. 53–54. H-1083, Hungary; 2 Research Group of Pediatrics and Nephrology, Hungarian Academy of Sciences, Budapest, Széchenyi István tér 9, Hungary; 3 Department of Laboratory Medicine, Semmelweis University, Budapest, Üllői út 26, Hungary; 4 Department of Measurement and Automation, Kálmán Kandó Faculty of Electrical Engineering, Óbuda University, Budapest Bécsi út 96/B, Hungary

**Keywords:** Perinatal asphyxia, Muscular artifact, Fourier analysis, EEG noise, aEEG

## Abstract

**Background:**

Amplitude-integrated electroencephalography (aEEG) is a useful tool to determine the severity of neonatal hypoxic-ischemic encephalopathy (HIE). Our aim was to assess the prevalence and study the origin of false normal aEEG recordings based on 85 aEEG recordings registered before six hours of age.

**Methods:**

Raw EEG recordings were reevaluated retrospectively with Fourier analysis to identify and describe the frequency patterns of the raw EEG signal, in cases with inconsistent aEEG recordings and clinical symptoms. Power spectral density curves, power (P) and median frequency (MF) were determined using the raw EEG. In 7 patients non-depolarizing muscle relaxant (NDMR) exposure was found. The EEG sections were analyzed and compared before and after NDMR administration.

**Results:**

The reevaluation found that the aEEG was truly normal in 4 neonates. In 3 neonates, high voltage electrocardiographic (ECG) artifacts were found with flat trace on raw EEG. High frequency component (HFC) was found as a cause of normal appearing aEEG in 10 neonates. HFC disappeared while P and MF decreased significantly upon NDMR administration in each observed case.

**Conclusion:**

Occurrence of false normal aEEG background pattern is relatively high in neonates with HIE and hypothermia. High frequency EEG artifacts suggestive of shivering were found to be the most common cause of false normal aEEG in hypothermic neonates while high voltage ECG artifacts are less common.

## Background

Recent clinical trials have provided clear evidence that therapeutic hypothermia initiated before six hours of age improves the neurological outcome in neonates with moderate hypoxic-ischemic encephalopathy (HIE) [1-4]. The early identification of neonates who may benefit from therapeutic hypothermia is of great importance. Amplitude-integrated electroencephalography (aEEG) is a tool that helps in the diagnosis of HIE [1,2,5,6]. The evaluation of aEEG recordings requires training and experience because several factors including muscular activity (movement, diaphragm spasm) or electrocardiographic (ECG) signal may affect the recordings [7-10]. Previously Hagmann et al. presented a case report of a neonate with characteristic signs and symptoms of HIE in the presence of normal appearing aEEG [8]. It is noteworthy that the aEEG became characteristic for HIE after administration of a non-depolarizing muscle relaxant (NDMR) agent, namely NDMR. This report confirms that muscular activity can contaminate the aEEG [8].

In the present retrospective study we investigated the prevalence and origin of normal appearing, false positive aEEG-s recorded in neonates with HIE.

## Methods

**Figure 1:**
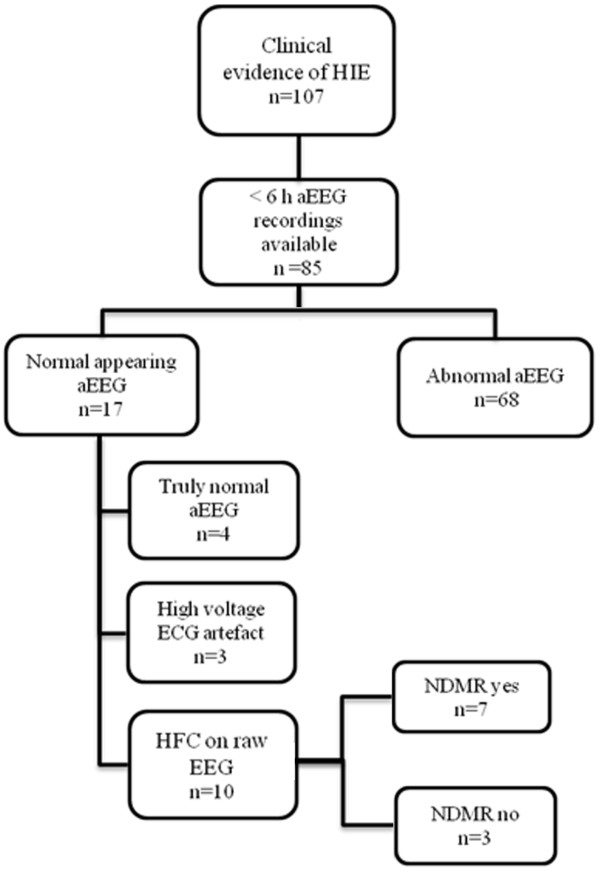
**Patient selection process.** Between 2006 and 2010 107 neonates fulfilled the clinical criteria for HIE. Of those, 85 neonates had aEEG recordings available within six hours of age. Seventeen aEEGs were reevaluated. In 4 cases raw EEGs were normal, 3 showed ECG artifacts. HFC were detected in 10 neonates and 7 received NDMR.

Medical records of 107 term neonates with moderate to severe HIE were reviewed (Figure 1). All neonates were outborn and admitted for hypothermia treatment (HT) to the level III NICU of the First Department of Pediatrics, Semmelweis University, Budapest, between 2006 January and 2010 July. The study was approved by the National Ethical Committee for Medical Research (TUKEB 6008/33/ETT/2002).

Criteria for HT were adopted from the TOBY trial [2]. Indication for HT included clinical criteria, neurological state and an abnormal aEEG trace suggesting moderate to severe HIE. Clinical criteria were at least one of the following: Apgar score ≤ 5 at 10 minutes after birth, continued need for resuscitation at 10 minutes after birth, pH < 7 and/or base deficit ≥ 16 mmol/l within the first hour of life. Neurological symptoms included altered level of consciousness and the presence of at least one of the following: muscular hypotonia, abnormal reflexes, absent or week suck, seizures.

Single channel EEG was recorded with electrode impedance below 2.5 kΩ, between two electrodes placed parietal on each side of the skull, and a third electrode served as a reference (Olympic CFM 6000). Criteria for normal aEEG trace named as continuous normal voltage (CNV) were continuous activity without seizure-like pattern with lower margin above 5 μV, and upper margin above 10 μV. All other aEEG background patterns were considered as abnormal. The aEEG classification terms were adopted from previous reports of Azzopardi et. al. and Hellstrom-Westas et. al. [2,11]. The primary bedside readers were well-trained attending physicians. In cases with contradictory aEEG findings and clinical symptoms, hypothermia was initiatiated at the discretion of the attending clinician. HT was started as early as possible (target was within 6 hours of life) using a water-filled mattress (Tecotherm®, TecCom, Halle, Germany), and was maintained for 72 hours. The target rectal temperature range was between 33-34°C. In the rewarming phase, temperature increase velocity was 0.5°C/h. All neonates were ventilated throughout the 72 hours of cooling and the rewarming phase.

As a first step of reevaluation the aEEG background activity was evaluated by a single expert reader (Anna Csekő) who was blinded to clinical history and symptoms of encephalopathy. Full aEEG recordings were reviewed and intervals with suspected artifacts or seizures were evaluated in detail using both aEEG and raw EEG signals. Criteria for electrical seizure: rhythmic, repetitive, stereotypic waveforms lasting 10 seconds on raw EEG.

**Figure 2:**
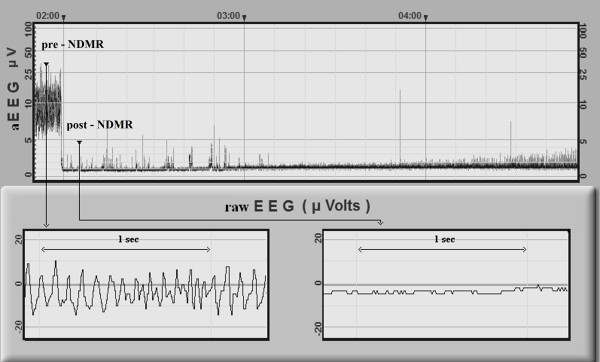
**Example of the changes on EEG following NDMR administration.** CNV aEEG background pattern turns into flat trace as a result of muscle relaxant. Bottom: HFC on raw EEG (left side) before NDMR and HFC disappeared upon medication, raw EEG without significant activity (right side).

**Figure 3:**
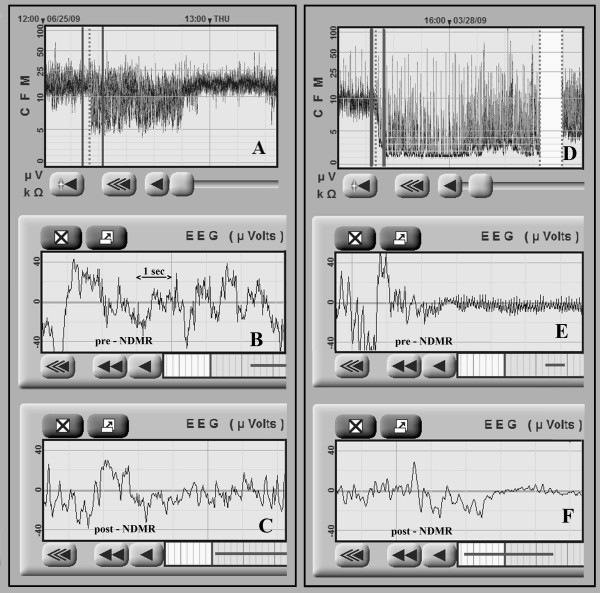
**High frequency component (HFC) on EEGs. A** and **D** shows normal like background pattern before NDMR administration. Pre-NDMR raw EEG shows HFC **(B,E)**, with delta, theta activity **(B)** and with a burst **(E)**. Post-NDMR EEG turned to abnormal, HFC disappeared **(C,F)**.

**Figure 4:**
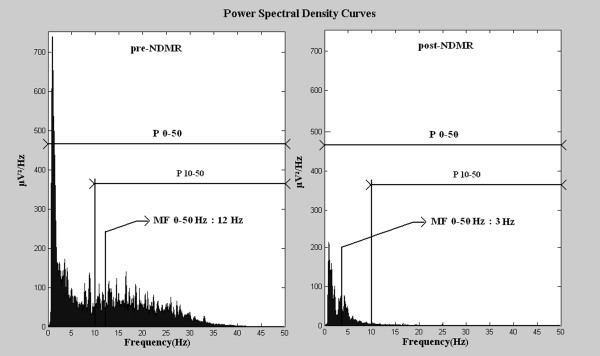
**Average PSD curves pre- and post-NDMR administration (n=7).** Power and median frequency decreased due to the medication. Power 10–50 is the area under power spectral density curve in this frequency range. Both left and right side from the median frequency the areas under curves are equal.

The second part of the study focused on a subpopulation of neonates with normal appearing aEEG background activity, which was inconsistent with the clinical signs and symptoms of encephalopathy. Detailed retrospective reevaluation of the raw EEG recordings with Fourier analysis was performed to identify and describe the frequency patterns of the raw EEG signal. Presence of dominant delta and theta waves was considered as normal, while presence of high frequency components (HFC) with or without delta and theta waves was suspected as false normal (Figures 2, 3). HFC was defined as at least 20 peaks per seconds with amplitudes above 5 μV on raw EEG, which refers to an activity above 10 Hz. In each case, a 10 minutes long digital raw EEG sample was exported and power spectral density (PSD) was computed by Fast Fourier Transformation [12,13] based on twenty randomly selected 6 seconds long periods. The EEG activity was filtered below 0.5 Hz. The power spectrum was limited to 0–50 Hz. In each case we determined the power (P) and the median frequency (MF) in the following frequency ranges: 0–50 Hz, 10–50 Hz. P is the area under PSD curves and MF is the frequency where the power is equal to 50 percent of P 0–50. For a more accurate interpretation see Figure 4. Data regarding NDMR administration, ordered by the attending clinician, were retrieved from the medical records. Samples of EEG recordings were exported exactly 5 minutes before and 5 minutes after the drug exposure and we compared pre- and post-NDMR data.

Based on the previous observation that the frequency of cold shivering closely correlates with body size, we used the formula: logY (Hz) = −0.18 logX(g) + 1.89 (where Y is the dominant frequency of cold shivering, X is the body weight and 1,89 is the correlation coefficient [14]) to determine the expected shivering frequency of the term human neonate.

For the analysis of EEG samples the software development environment was MATLAB R2008b. For statistical analysis Statistica 8 was used: Wilcoxon matched pair test was performed to evaluate the effect of NDMR. P < 0.01 was considered statistically significant.

## Results

Eighty-five neonates had aEEG recordings before six hours of age, 68 (80%) had an aEEG recording with an abnormal background activity that was in agreement with the clinical signs and symptoms. In 17 (20%) neonates, aEEG recordings were of normal appearance and they were inconsistent with the clinical signs and symptoms. Of the 17 neonates, three presented with high voltage electrocardiographic artifacts with flat trace on raw EEG; their data were excluded from further analyses. In 4 cases, EEG background patterns were normal based on the presence of theta and delta waves on raw EEG and the result of Fourier analysis. HFC indicating false normal aEEG was identified in 10 neonates [in nine cases Synchronized Intermittent Mandatory Ventilation mode was applied and in one High Frequency Oscillatory Ventilation was applied (f = 10 Hz)], in each case the high frequency artifact was visible on the raw EEG. Seven of these neonates were exposed to NDMR, either one dose of pancuronium (0.1 mg/kg BW), or vecuronium (0.1 mg/kg BW) or atracurium (0.3 mg/kg BW). Two neonates were pre-treated with morphine (0.1 mg/kg BW) and one with phenobarbitone (20 mg/kg BW) at least 2 hours before NDMR administration, while two neonates were on continuous morphine infusion (10 μg/kg BW/h). In neonates with HFC (n = 10) we found that 39% of the total of P 0–50 originated from frequency ranges above 10 Hz. In NDMR treated neonates (n =7) we observed a sharp reduction in the aEEG trace following NDMR administration. In each neonate the continuous normal voltage aEEG background pattern immediately became abnormal upon NDMR administration (flat trace, burst-suppression and discontinuous normal voltage pattern, as observed in 5, 1 and 1 patient, respectively). All P 0–50, P 10–50, MF 0–50 and MF 10–50 values were reduced after NDMR administration (p < 0.001, Table 1). The proportion of P 10-50/P 0–50 between pre- and post-NDMR raw EEGs revealed that the marked decline of P 0–50 is dominantly due to the reduction of power in the frequency range above 10 Hz. The median of the MF 10–50 values was 18 Hz (Table 1). Calculated cold shivering frequency for our neonates was 18 Hz.

**Table 1:** Calculated data from power spectral density curves

	**Normal EEG (n = 4)**	**False normal EEG (n = 10)**		
		**Pre-NDMR**	**Post-NDMR (n = 7)**	
P 0-50	4297 (3073, 7160)	1381 (683, 2471)	77 (39, 118)	p < 0.001
P 10-50	370 (240, 649)	538 (305, 966)	12 (8, 22)	p < 0.001
P 10-50/P 0-50	0.09	0.39	0.16	
MF 0-50	2 (1.5, 2.5)	9 (3, 16)	3 (2, 5)	p < 0.001
MF 10-50	15 (13, 18)	18 (16, 20)	11 (10, 12)	p < 0.001

Power, P(μV^2^); Median frequency, MF(Hz), median (25th, 75th). The p shows the statistical effect of NDMR on P 0–50, P 10–50, MF 0–50, MF 10–50.

One of the 17 neonates had electroencephalographic seizures 2 hours after the NDMR administration.

The 72 hours HT was not applied in 5 cases from the studied 17 neonates, however, most neonates were hypothermic at the time of the observation because of permissive or passive cooling prior admission. Clinical data including core temperature at the time of observation of the 17 neonates with normal appearing aEEG are presented in Table 2.

**Table 2:** Clinical data of HIE infants with normal appearing aEEG

Gestational age (weeks)	38 (36–41)
Birth weight (gram)	3393 (2710–5390)
Female/Male	6/11
Apgar 1/10 (median)	2/6
pH (<1 hour)	6.9 (6.6-7.28)
Base deficit (mmol/l)	17 (7–26)
Time of the sampling (hour)	3 (2–6)
Core temperature (°C)	34.5 (32.2-38.2)
Mean (min - max)	

## Discussion

In the current study we observed that aEEG done before six hours of age may show a normal like pattern in a relatively high proportion (15%) of term neonates presenting with clinical signs and symptoms of moderate to severe HIE and subjected to hypothermia.

Previously the occurrence rate of normal aEEG in neonates with encephalopathy was reported to be around 10% [5,15]. Rooij et al. demonstrated adverse long term outcome in 9% of those neonates who had history of asphyxia and normal aEEG background pattern recorded before the 6th hour of life [16]. This phenomenon is relatively frequent under HT, however, no analysis was made to assess the possible explanation.

The healthy term neonate’s brain electric activity is mostly below 4 Hz (characterized by predominance of delta and theta waves, and there is little activity above 10 Hz) [13,16]. Therefore, in the present study, we used 10 Hz as a cut-off value to distinguish between cerebral and non-cerebral activity. The frequency range of muscular activity is up to 200 Hz [17]. The amplitude of muscular electric activity is three magnitude larger than that of brain electric activity (mV versus μV), thus it seems likely that muscular electric activity interferes with the aEEG recording [18]. The disappearance of HFC in NDMR exposed neonates emphasizes the role of muscular activity, namely we presume that the HFC was a high frequency muscular artifact (HFMA).

The recognition of HFMA is usually easy, but it is more challenging when delta or theta activity is present on the raw EEG [for example our moderately abnormal, discontinuous background pattern could be missed without NDMR administration (Figure 3)].

Neonatal seizures are associated with increased mortality. It is important to distinguish between seizure and non-seizure activity, however, some artifacts may mimic electroencephalographic seizures (e.g. pulse artifact). The HFMA usually presents as a rhythmic artifact on the raw EEG, and raw EEG on fast paper speed (60 mm/sec or higher) may lead to misinterpretation, especially when theta or delta waves are missing (Figure 2, pre-NDMR). Although there are some features which could help at the bedside: (1) the frequency of HFMA is usually higher, (2) the amplitude of the HFMA is usually lower.

Muscular shivering frequently occurs during hypothermia. Under hypothermic conditions the neonate produces heat both via non-shivering thermogenesis (increased catabolism) and involuntary muscular tonic or rhythmic tremors known as shivering [19]. Unfortunately, data regarding the frequency pattern of neonatal muscle activity during cold shivering is not available. The fact that the estimated shivering frequency and the measured MF 10–50 values were the same supports the assumption that shivering might play a role in the development of the HFMA in our cohort. Furthermore, the low actual body temperature at the time of the observation may have provoked shivering in the neonates; however, the shivering was not documented clinically in any case.

### Limitations

EMG was not recorded simultaneously with aEEG, which could have directly confirmed our hypothesis that the cold shivering caused the HFMA. In many departments EMG is not an easily accessible examination in the early postnatal hours and under emergency conditions. In addition, conventional EEG as a gold standard method could strengthened our observations.

Furthermore, prior CNS drug administration theoretically might have altered our observations in some neonates [20]. However, CNS drugs were unlikely to have influenced our results as we compared pre- and post-NDMR EEG recordings within a relatively short observation period when CNS drug regimen was not altered. The relatively low number of patients exposed to CNS drugs resulting in insufficient analgesia may have increased the risk for the development of shivering in our hypothermic neonates.

## Conclusions

Our results are consistent with earlier observations that muscular activity may bias the evaluation of aEEG in some asphyxiated neonates [8]. Further research is warranted on the significance and appropriate recognition of hypothermia induced shivering in neonates under HT. The parallel evaluation of raw EEG and aEEG could decrease the misinterpretation of aEEG.

## Abbreviations

aEEG: Amplitude-integrated electroencephalogram; BW: Body weight; CNS: Central nervous system; CNV: Continuous normal voltage; ECG: Electrocardiography; EEG: Electroencephalogram; EMG: Electromyography; HFC: High frequency component; HFMA: High frequency muscular artifact; HIE: Hypoxic ischemic encephalopathy; HT: Hypothermia treatment; MF: Median frequency; NDMR: Non-depolarizing muscle relaxant agent; NICU: Neonatal intensive care unit; P: Power; PSD: Power spectral density.

## Competing interests

The authors declare that they have no competing interests.

## Authors’ contributions

GM was responsible for patient screening, and EEG analysis, statistical analysis and writing the manuscript and approved the final manuscript as submitted. BV carried out the initial analyses, reviewed and revised the manuscript and approved the final manuscript as submitted. AC participated in patient selection, clinical data collection, aEEG analysis and revised the manuscript and approved the final manuscript as submitted. DZ participated in the data collection and revised the manuscript and approved the final manuscript as submitted. GS participated in the Fourier analysis and revised the manuscript and approved the final manuscript as submitted. MS was responsible for protocol development, patient screening, enrollment, preliminary data analysis and writing the manuscript and approved the final manuscript as submitted.

## Authors’ information

Gábor Marics (MD and electrical engineer) is a PhD student at First Department of Pediatrics, Semmelweis University. Barna Vásárhelyi MD PhD is the head of the Department of Laboratory Medicine, Semmelweis University. Anna Csekő MD is a fellow at First Department of Pediatrics, Semmelweis University. Dávid Zakariás is a 6th year medical student, Semmelweis University. György Schuster PhD is the head of the Department of Measurement and Automation, Óbuda University. Miklós Szabó MD PhD is the head of the NICU at first Department of Pediatrics, Semmelweis University.

## Pre-publication history

The pre-publication history for this paper can be accessed here:

http://www.biomedcentral.com/1471-2431/13/194/prepub
